# Development of an automated machine learning-based prognostic prediction model for emergency septic shock patients

**DOI:** 10.3389/fmed.2026.1860425

**Published:** 2026-10-26

**Authors:** Suxiang Wei, Haiou Chen, Xueyan Li, Tingting Ke, Xinyi Chen, Caili Lin, Limin Qian, Xiusong Wang

**Affiliations:** Emergency Department, The First Affiliated Hospital of Wenzhou Medical University, Wenzhou, China

**Keywords:** automated machine learning (AutoML), clinical decision support system, explainable artificial intelligence, prognostic prediction, septic shock

## Abstract

**Objective:**

To overcome the limitations of static assessment, linear modeling, and lack of individualization in traditional scoring systems for prognostic evaluation of emergency septic shock patients, we developed an admission-based risk prediction model with real-time calculation capability to achieve precise stratification of 28-day all-cause mortality risk using initial clinical presentations.

**Methods:**

We retrospectively enrolled 913 septic shock patients admitted to a tertiary hospital between January 2019 and January 2025. An AutoML framework was constructed using an Improved Red Fox Optimization (IRFO) algorithm, which optimized feature selection and hyperparameter tuning through chaotic mapping initialization and dynamic Lévy flight strategies. The dataset was split into training (*n* = 730, 80%) and testing (*n* = 183, 20%) sets. Six benchmark models were synchronously developed for comparison, including Logistic Regression (LR), Support Vector Machine (SVM), Adaptive Boosting (AdaBoost), Extreme Gradient Boosting (XGBoost), and Light Gradient Boosting Machine (LightGBM). Synthetic Minority Over-sampling Technique (SMOTE) addressed class imbalance, and model performance was evaluated via five-fold cross-validation using key metrics (AUC, sensitivity, specificity, Brier score). Clinical utility was validated via Decision Curve Analysis (DCA) and SHapley Additive exPlanations (SHAP) interpretability models.

**Results:**

In the training set cross-validation, the AutoML model exhibited robust discriminative capacity (ROC-AUC=0.9127, PR-AUC=0.9059). When applied to the independent test set, it maintained optimal performance: ROC-AUC=0.8911, PR-AUC=0.8790. At the default threshold of 0.5, the model achieved sensitivity=0.9022 and specificity=0.7143; at the clinically recommended operating threshold of 0.35, sensitivity increased to 0.951 (specificity:0.648) to prioritize minimizing missed fatalities. Critical predictive features identified included Neutrophil-to-Lymphocyte Ratio (NLR), Hemoglobin (Hb), Procalcitonin (PCT), D-dimer, Mean Platelet Volume (MPV), and age. Decision Curve Analysis demonstrated significantly superior net clinical benefit over conventional intervention strategies when risk threshold exceeded 10%. For the test set, the established AutoML model demonstrated significantly superior predictive performance compared to conventional scores (SOFA, APACHE II, and qSOFA), highlighting substantially enhanced predictive accuracy.

**Conclusion:**

The IRFO-optimized AutoML framework constructed a high-accuracy, interpretable prognostic model for septic shock based on admission routine blood parameters, capturing nonlinear interactions that traditional linear approaches may overlook. On the independent test set, the model demonstrated favorable predictive performance compared to traditional scoring systems (SOFA, APACHE II, qSOFA), and the accompanying visualized decision-support interface offers a potentially useful tool for emergency risk stratification.

## 1 Introduction

Sepsis is a life-threatening organ dysfunction caused by a dysregulated host response to infection (1, 2). Septic shock, characterized by concurrent circulatory and metabolic dysfunction, represents one of the leading causes of mortality among intensive care unit (ICU) patients worldwide (3, 4). Early detection and intervention are critical for improving patient prognosis. Despite significant advancements in diagnosis and treatment following the “Surviving Sepsis Campaign”, both incidence and mortality rates remain persistently high, establishing sepsis as a primary risk factor for in-hospital death and 30-day readmission (5–7). The identification of rapid, accessible, and effective diagnostic markers for risk stratification in emergency critically ill patients could substantially improve survival rates (8). Traditional scoring systems such as the Sepsis-related Organ Failure Assessment (SOFA), Acute Physiology and Chronic Health Evaluation II (APACHE II), quick SOFA (qSOFA), and Modified Early Warning Score (MEWS) are clinically employed to predict mortality in multi-organ dysfunction (9, 10). However, these demonstrate limited utility for real-time emergency assessment. Emerging sepsis biomarkers in early research phases face implementation barriers in developing countries due to prohibitive costs (11). Given sepsis’ temporally dynamic host response and immunological complexity, single biomarkers exhibit limited prognostic efficacy (12). Increasing attention has focused on routine blood parameters, though their clinical utility remains controversial. The Neutrophil-to-Lymphocyte Ratio (NLR) serves as a validated prognostic indicator for malignancies, heart failure, and other conditions (13–15). Additional parameters including Procalcitonin (PCT), Hemoglobin (Hb), D-dimer, Mean Platelet Volume (MPV), and MPV/platelet ratio (MPR) have demonstrated sepsis prognostic relevance (16, 17).

Nevertheless, traditional scoring systems and biomarker-based models face three critical limitations in emergency septic shock prognosis: (1) Static constraints: Models predominantly rely on admission or single-time-point data, failing to dynamically capture sepsis pathophysiological evolution and therapeutic responses. (2) Feature simplification and nonlinearity neglect: Linear modeling approaches with preset weights cannot integrate multidimensional clinical-laboratory parameters. Sepsis inherently involves complex nonlinear interactions across immune, coagulation, and metabolic systems—patterns disregarded by conventional models. (3) Insufficient feature mining and personalization gaps: While parameters like Hb, D-dimer, and MPR show potential, the deep predictive value embedded within readily available, low-cost routine indicators remains underexploited, impeding personalized prognostic assessment.

The emergence of Automated Machine Learning (AutoML) offers transformative solutions by automatically processing high-dimensional data, detecting complex nonlinear feature interactions beyond linear model capabilities, identifying optimal feature combinations from routine data, and incorporating time-series parameters to reflect disease progression. Consequently, this study aims to overcome static scoring system limitations by leveraging AutoML's pattern recognition power to develop a rapid, precise, bedside mortality risk prediction tool for emergency septic shock patients, enabling immediate risk stratification upon admission.

## 2 Methods

### 2.1 Study population

This retrospective study enrolled 913 patients diagnosed with septic shock at a tertiary hospital in Zhejiang Province, China between January 2019 and January 2025 after applying inclusion and exclusion criteria. The dataset was partitioned into training (*n* = 730, 80%) and testing sets (*n* = 183, 20%) in an 8:2 ratio. Ethical approval was granted by the Institutional Review Board (Approval No: KY2025-R389) with waived informed consent given the retrospective design. Eligible patients met established septic shock diagnostic criteria (1). Exclusion criteria comprised: age below 18 years; pregnancy; acute cerebrovascular disease or coronary syndrome; contraindications to central venous catheter placement; chronic liver or kidney dysfunction; immunologic disorders or malignancy; requirement for immediate surgical intervention; inter-hospital transfer within 6 hours of emergency admission; hematologic diseases (including lymphoma and leukemia); pre-admission trauma or active bleeding events (e.g., gastrointestinal hemorrhage) during hospitalization; pre-hospital blood product transfusion (to mitigate confounding effects on hemoglobin and related parameters); treatment withdrawal decisions; and hospital-acquired septic shock episodes. Sample inclusion details are detailed in Figure 1.

**Figure 1:**
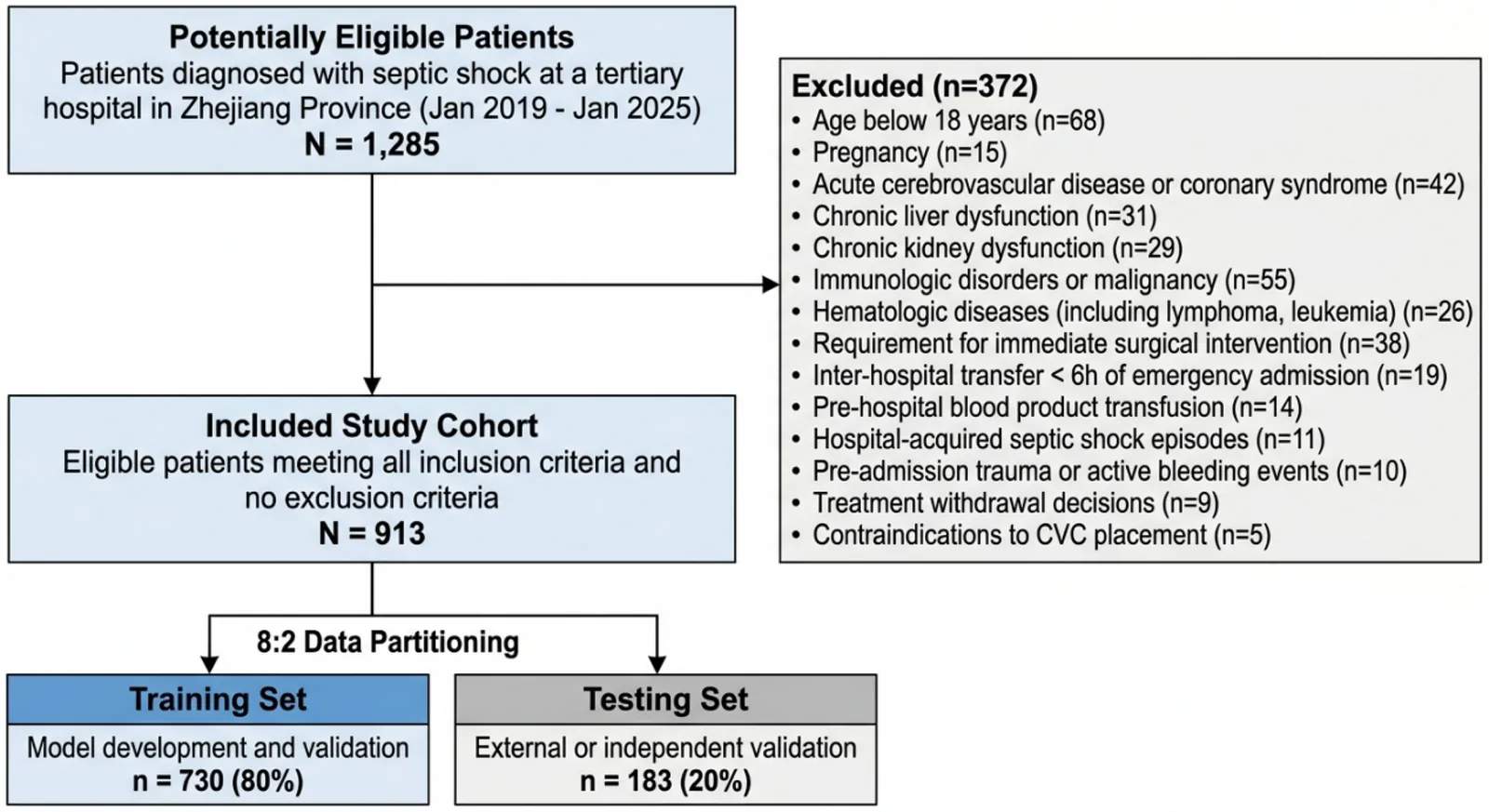
Flow chart of sample inclusion.

Additionally, 169 new patients consecutively admitted to the same hospital from February to December 2025 were enrolled as a temporal validation cohort. This additional cohort did not overlap with the original cohort and was not involved in model development, and a total of 1082 patients were included across the two study phases. The final AutoML model constructed based on the original cohort was frozen and directly applied to the new temporal validation cohort, without retraining, feature reselection, hyperparameter tuning, or threshold optimization.

### 2.2 Data collection

Data were extracted from the electronic medical records of 913 unique patients meeting septic shock diagnostic criteria. To ensure patient uniqueness, we implemented a multi-identifier verification system combining patient name, hospital admission ID, and the precise timestamp of emergency department registration. Duplicate entries—defined as identical identifiers across multiple admissions—were excluded at the data extraction phase. Electronic medical records provided comprehensive data extraction covering clinical baseline characteristics, laboratory parameters, and outcome measures. Baseline assessments included age, sex, body mass index (BMI), medical history, and septic shock etiology. Laboratory indices measured at admission encompassed base excess (BE), albumin (ALB), white blood cell count (WBC), neutrophil count (NEU), monocyte count (MON), lymphocyte count (LYM), platelet count (PLT), mean platelet volume (MPV), hemoglobin (Hb), C-reactive protein (CRP), D-dimer, procalcitonin (PCT), neutrophil-to-lymphocyte ratio (NLR), and monocyte-to-lymphocyte ratio (MLR). All laboratory indicators were measured within <2 hours post-admission from blood drawn during initial emergency assessment to capture physiological states at decisive clinical intervention points. It should be emphasized that blood samples for all laboratory indices were collected immediately after emergency triage and before the initiation of any resuscitation or drug therapy, rather than after initial stabilization. Blood collection time points were consistent across patients, thereby ensuring that the measured values reflected the baseline physiological state before therapeutic intervention and avoiding confounding of these indices caused by interventions such as fluid resuscitation, vasoactive agents, or antibiotics. The primary clinical endpoint was 28-day all-cause mortality. Patients withdrew from treatment before 28-day outcome assessment (e.g., early discharge against medical advice, care limitation decisions) were prospectively excluded during data screening. This ensured 100% 28-day mortality ascertainment via institutional electronic records linkage for all analyzed cases. Consequently, our cohort exclusively comprised patients with complete 28-day outcomes, eliminating loss-to-follow-up bias.

The collected clinical and laboratory data underwent systematic preprocessing prior to model analysis. For missing values in continuous variables, imputation was performed based on distributional characteristics: mean imputation was applied for variables conforming to or approximating normal distribution, while median imputation was used for those exhibiting skewed distribution. For categorical variables, mode imputation was employed. All core predictors included in this study demonstrated missing rates below 5%, and the aforementioned imputation methods showed no significant impact on the overall data distribution. All data processing procedures were completed before model training.

### 2.3 Automated machine learning modeling pipeline

To construct a robust and information-leakage-free prognostic prediction model, this study designed and implemented a rigorous double-layer nested cross-validation automated machine learning (AutoML) framework, with the core being the Improved Rüppell’s Fox Optimizer (IRFO) algorithm.

#### 2.3.1 Improved Rüppell's Fox Optimizer (IRFO)

The original Rüppell's Fox Optimizer (RFO) is a metaheuristic algorithm that simulates fox foraging behavior. It was enhanced to IRFO by introducing a chaotic mapping initialization strategy to optimize the distribution of the initial population and employing dynamic Lévy flight strategies to balance exploration-exploitation efficiency. The optimization performance of IRFO has been validated on CEC2022 benchmark test functions. To facilitate reproducibility, the pseudocode of the IRFO algorithm, illustrating its dual-task optimization logic for feature selection and hyperparameter tuning within the AutoML framework, is detailed in Supplementary Material 1.

The comprehensive search space designed for the IRFO-driven AutoML encompasses a pre-defined library of candidate features and a repository of models with their corresponding hyperparameter ranges. The model repository for inner-loop optimization comprised XGBoost and LightGBM classifiers, whose hyperparameter search domains are detailed in Table 1. During each iteration, IRFO simultaneously selects a feature subset from the candidate pool and identifies the optimal hyperparameter set for the chosen base learner, guided by the objective of maximizing the 5-fold cross-validated AUC on the training fold.

**Table 1:** AutoML model repository and hyperparameter search space.

Model	Hyperparameter	Type	Search Range	Step/Scale
XGBoost	n_estimators	Integer	[50, 500]	10
	max_depth	Integer	[3, 15]	1
	learning_rate	Continuous	[0.01, 0.3]	Log-uniform
	subsample	Continuous	[0.5, 1.0]	Uniform
	colsample_bytree	Continuous	[0.5, 1.0]	Uniform
LightGBM	n_estimators	Integer	[50, 500]	10
	num_leaves	Integer	[15, 255]	5
	learning_rate	Continuous	[0.01, 0.3]	Log-uniform
	feature_fraction	Continuous	[0.5, 1.0]	Uniform
	min_child_samples	Integer	[5, 100]	5

#### 2.3.2 Nested cross-validation modeling pipeline

The entire modeling pipeline strictly isolates model development and final evaluation phases to ensure the test set remains unused until model finalization. Specifically, the total dataset was randomly divided into an independent, locked test set (*n* = 183) and a training-development set (*n* = 730) at an 8:2 ratio. This test set was kept isolated throughout subsequent model optimization and selection steps, solely for final model performance assessment. The outer loop (performance evaluation) employed five-fold cross-validation on the training-development set, where each fold iteration subdivided the data into a “training fold” and a “validation fold.” On each “training fold” from the outer iteration, the inner loop (model optimization) executed IRFO-driven AutoML inner-layer optimization. IRFO performed a dual task: (a) feature subset selection in discrete space to screen the most predictive combinations from all candidate features; and (b) hyperparameter tuning in continuous space for selected machine learning models (e.g., base learners such as XGBoost and LightGBM), with the objective of minimizing the target function (e.g., negative AUC) computed via cross-validation on the “training fold”. Using the optimal feature subset and hyperparameter configuration determined by the inner loop, the model was retrained on the entire “training fold” and its performance was evaluated on the independent “validation fold,” with evaluation metrics recorded for that fold. Steps 2–4 were repeated to complete the five-fold outer loop, resulting in five sets of optimal model configurations and performance estimates from their respective validation folds. To obtain a stable final model, a consensus feature set (commonly selected features across the majority of folds) derived from the five-fold optimization was applied. Then, an integrated AutoML model was retrained on the entire training-development set (*n* = 730) using the median or mode values of the five-fold hyperparameters. For final evaluation, the integrated AutoML model finalized on the full training-development set in Step 5 was applied to the fully independent test set (*n* = 183), with all reported performance metrics (e.g., AUC, sensitivity, specificity, Brier score) calculated. This step provided an unbiased estimation of model generalization capability. Additionally, as a comparison, five benchmark models–Logistic Regression (LR), Support Vector Machine (SVM), Adaptive Boosting (AdaBoost), Extreme Gradient Boosting (XGBoost), and Light Gradient Boosting Machine (LightGBM)—were established synchronously. All models were trained and compared under the identical nested cross-validation framework and used standardized processed data, with this computational workflow depicted in Figure 2.

**Figure 2:**
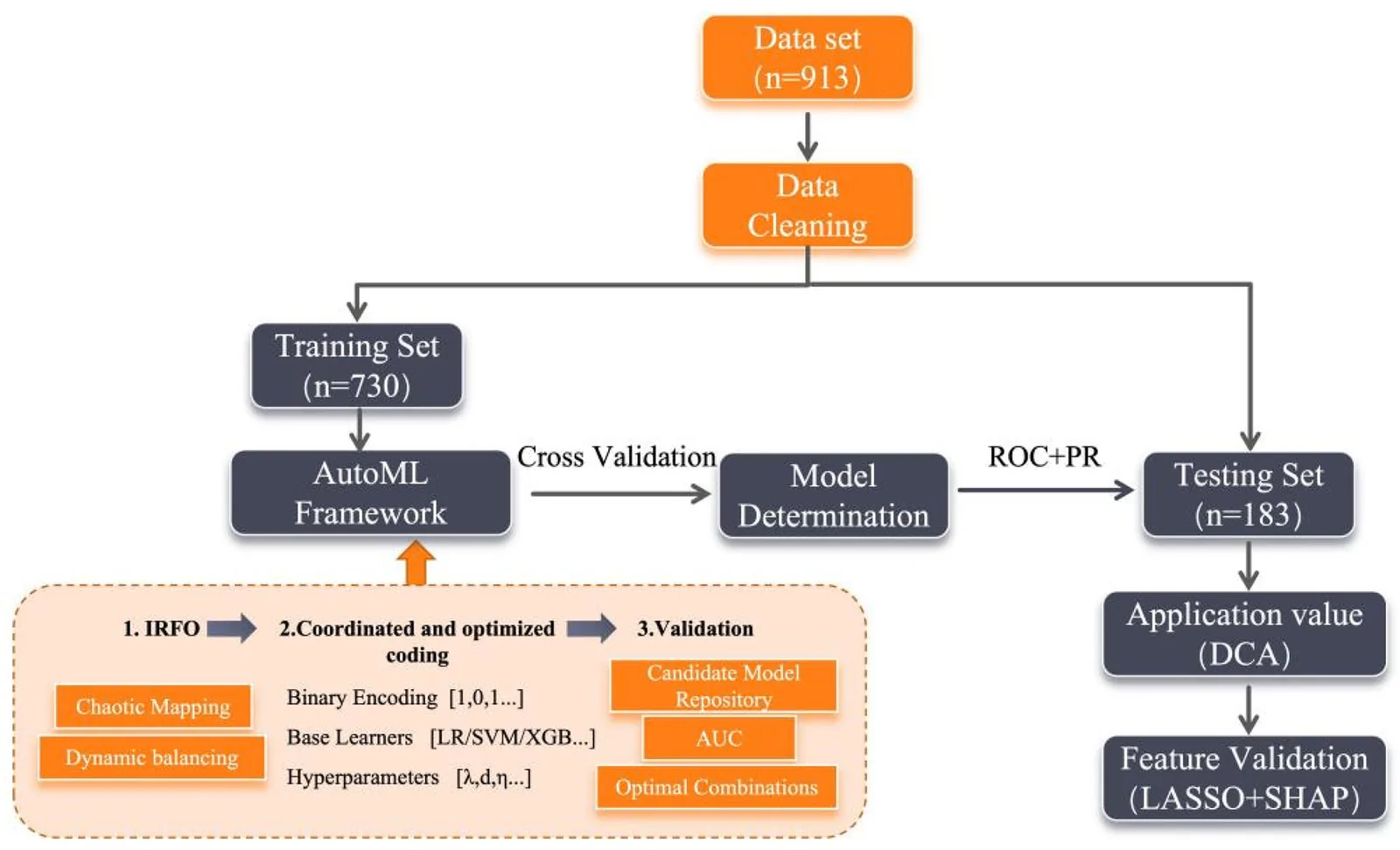
Study flowchart.

### 2.4 Evaluation metrics

A multidimensional evaluation system was established with three components. Classification performance was assessed through Accuracy (ACC), Sensitivity (SEN), Specificity (SPE), F1-score, Area Under the ROC Curve (ROC-AUC), and Area Under the Precision-Recall Curve (PR-AUC) to holistically evaluate model discriminative capability under class imbalance. Calibration performance was measured using calibration curves and Brier scores, where lower values indicate superior probabilistic accuracy. Clinical utility was quantified via Decision Curve Analysis (DCA), which calculates net benefit (NB) across risk thresholds using the formula:

$$NB=\frac{TP}{N}-\frac{FP}{N}\times \frac{\;p_{t}}{1-p_{t}}$$

where *TP* is true positives, *FP* is false positives, *N* is total sample size, and *pt* is the risk threshold. By comparing *NB* with reference lines for traditional intervention strategies, the effective interval for model-assisted decision-making was validated.

### 2.5 Interpretability analysis

A three-stage interpretability workflow was implemented: first, the AutoML framework pre-selected prognostic features through predefined search spaces and optimization targets; subsequently, LASSO regression analysis with L1 regularization verified feature robustness, sparsity, and stability against overfitting, enabling comparison between AutoML-selected and LASSO-validated features; finally, Shapley Additive exPlanations (SHAP) based on cooperative game theory quantified global feature importance, visualized prediction logic, and validated clinical plausibility through feature contribution analysis.

### 2.6 Statistical analysis

MATLAB 2024a facilitated predictive modeling while SPSS 26.0 performed statistical analyses. Continuous variables were expressed as mean ± standard deviation (SD) for normally distributed data or median with interquartile range (IQR) for non-normally distributed data; categorical variables were presented as frequencies (percentages). Intergroup comparisons applied Student's t-test for normally distributed continuous variables, Mann–Whitney U test for non-normal continuous variables, and Pearson's *χ*^2^ test for categorical variables following normality assessment. Statistical significance was defined as two-tailed *P* < 0.05 (*α*=0.05), with results tabulated for structured presentation.

## 3 Results

### 3.1 Baseline characteristics

The study enrolled 913 patients with septic shock, including 458 non-survivors (50.16% 28-day mortality). No statistically significant differences existed between training (*n* = 730) and testing (*n* = 183) sets across all baseline characteristics and laboratory parameters (all *P* > 0.05). Stratified randomization was effective, with comparable 28-day mortality proportions (training 50.3% vs testing 49.7%; *χ*^2^ = 0.021, *P* = 0.884), confirming comparable cohort distributions as detailed in Table 2.

**Table 2:** Comparison of clinical characteristics between training set and testing set.

Feature	Training Set (*n* = 730)	Testing Set (*n* = 183)	Statistic	*P*-value
Age [years, Median (Q₁, Q₃)]	67.0 (59.0, 75.0)	65.0 (58.0, 74.0)	−0.852	0.397
Sex [Male, n (%)]	418 (57.3%)	102 (55.7%)	0.152	0.697
BMI [kg/m^2^, Median (Q₁, Q₃)]	25.0 (22.7, 27.9)	25.2 (22.9, 28.1)	−0.365	0.715
Respiratory Rate (RR) [breaths/min, Median (Q₁, Q₃)]	22.0 (18.0, 26.0)	23.0 (18.0, 26.0)	−0.923	0.356
Laboratory Parameters [Median (Q₁, Q₃)]				
BE (mmol/L)	−3.8 (−6.2, −1.5)	−4.0 (−6.5, −1.8)	−0.634	0.526
ALB (g/L)	31.5 (28.0, 35.0)	32.0 (28.0, 35.0)	−0.412	0.680
CRP(mg/L)	95.0 (53.0, 152.0)	92.0 (52.0,148.0)	0.550	0.582
PCT(g/L)	7.8 (2.9, 16.5)	7.5 (2.7, 16.0)	0.177	0.860
Ferritin(μg/L)	780 (380, 1500)	750 (360, 1450)	0.322	0.747
D-Dimer(μg/L)	2150 (1180, 4080)	2050 (1120, 4000)	0.261	0.794
WBC(×10⁹/L)	12.5 (8.5, 17.0)	12.2 (8.7, 16.5)	0.238	0.812
NEU(×10⁹/L)	10.8 (7.5, 14.2)	10.6 (7.3, 14.0)	0.317	0.751
MON(×10⁹/L)	0.55 (0.37, 0.80)	0.58 (0.38, 0.82)	−0.417	0.676
LYM(×10⁹/L)	0.85 (0.55, 1.15)	0.88 (0.56, 1.18)	−0.931	0.352
NLR	12.7 (8.3, 18.0)	12.5 (8.1, 17.8)	0.082	0.935
MLR	0.68 (0.43, 0.95)	0.67 (0.44, 0.95)	0.138	0.890
MPV(fL)	10.7 (9.8, 11.6)	10.6 (9.7, 11.5)	0.351	0.726
PLT(×10⁹L)	158 (106, 224)	154 (104, 218)	0.271	0.787
Hb(g/L)	100 (88, 114)	102 (90, 115)	−0.973	0.331
Comorbidities [n (%)]				
Diabetes Mellitus	175 (24.0%)	41 (22.4%)	0.198	0.656
Hypertension	306 (41.9%)	79 (43.2%)	0.090	0.764
Coronary Heart Disease	138 (18.9%)	34 (18.6%)	0.012	0.914
COPD	102 (14.0%)	25 (13.7%)	0.011	0.917
Infection Site [n (%)]				
Pulmonary	382 (52.3%)	96 (52.5%)	0.002	0.968
Abdominal	146 (20.0%)	35 (19.1%)	0.074	0.785
Urinary Tract	109 (14.9%)	29 (15.8%)	0.106	0.745
Polymicrobial Infections	66 (9.0%)	16 (8.7%)	0.020	0.887
Other	27 (3.7%)	7 (3.8%)	0.008	0.930

### 3.2 Algorithm optimization performance

To validate IRFO's optimization capability, comparative experiments against original RFO, Whale Optimization Algorithm (WOA), Grey Wolf Optimizer (GWO), Particle Swarm Optimization (PSO), Genetic Algorithm (GA), GA-PSO, and GA-ACO were conducted using all 12 CEC2022 benchmark functions. With dimension=10, population size=30, and maximum iterations=500 (30 independent runs), boxplots demonstrated IRFO's superior stability across most functions. IRFO significantly outperformed comparators in solution quality robustness (Supplementary Figure S1). Convergence curve analysis further confirmed IRFO's accelerated convergence rate and reduced local optimum susceptibility (Supplementary Figure S2).

### 3.3 Model training performance

Six machine learning models were systematically evaluated on the training set. AutoML demonstrated optimal performance with ROC-AUC=0.9127 and PR-AUC=0.9059. Its exceptional F1-score (0.8388) highlighted superior precision-recall balance for clinical deployment (Table 3). Age, NLR, MPV, Hb, PCT, and D-dimer were identified as top predictive features.Visual evidence is presented in Figure 3: Panel A (ROC curves) and Panel B (PR curves) collectively confirm AutoML's discriminative advantage.

**Table 3:** Cross-validation prediction efficacy metrics on the training set.

Models	PRE	SEN	SPE	ACC	F1	ROC-AUC	PR-AUC
LR	0.6803	0.8607	0.5934	0.7274	0.7600	0.8042	0.7826
SVM	0.6556	0.8634	0.5440	0.7041	0.7453	0.7824	0.7767
Adaboost	0.7237	0.8661	0.6676	0.7671	0.7886	0.8443	0.8242
XGBoost	0.8292	0.8224	0.8297	0.8260	0.8258	0.8997	0.8978
LightGBM	0.7902	0.8852	0.7637	0.8247	0.8351	0.9021	0.8994
AutoML	0.8060	0.8743	0.7885	0.8315	0.8388	0.9127	0.9059

**Figure 3:**
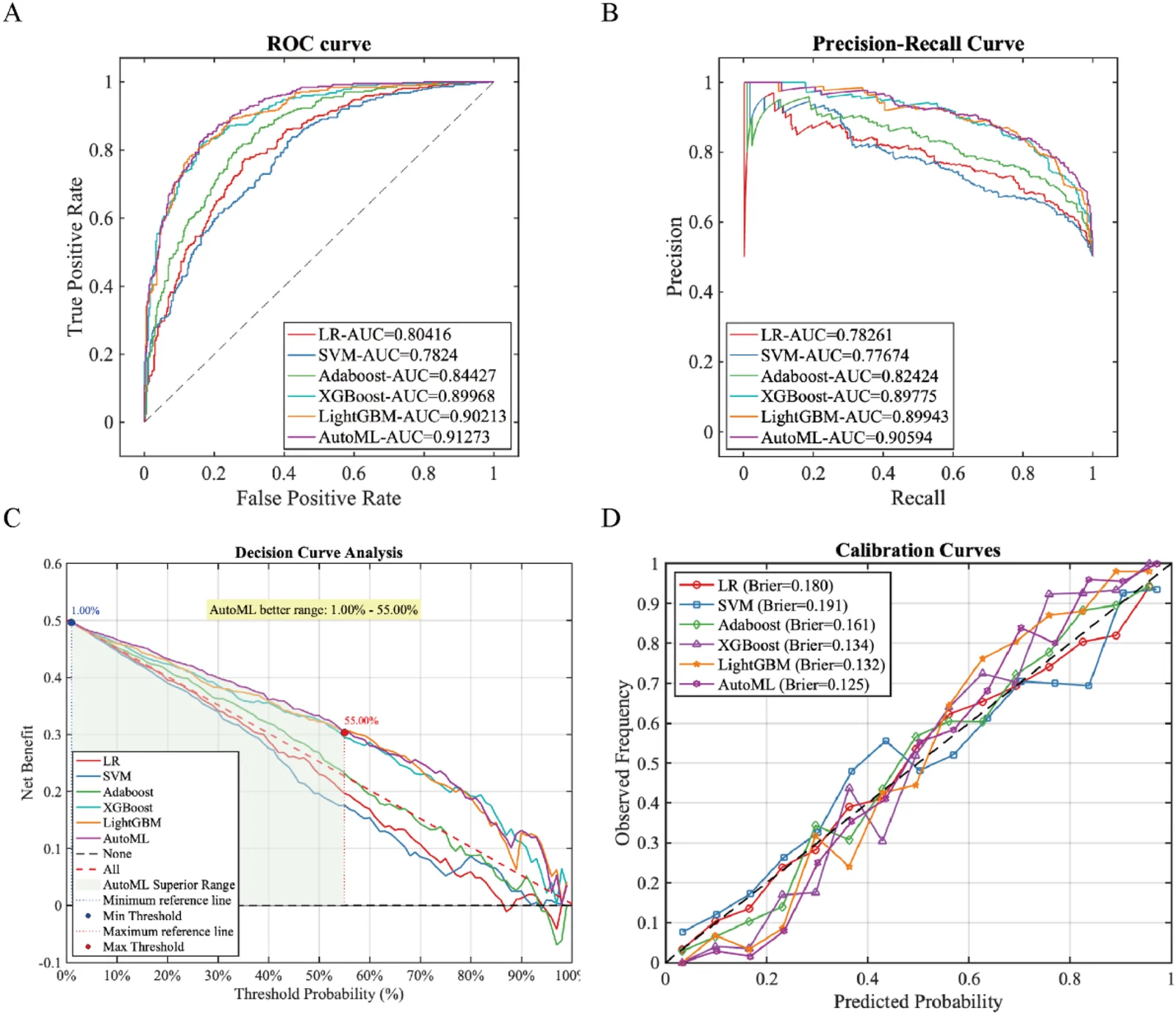
Training set cross-validation performance. **(A)** ROC curves; **(B)** PR curves; **(C)** DCA curves; **(D)** Calibration curves.

DCA demonstrated significant net clinical benefit for AutoML across the 1%-55% threshold probability range (Figure 3C), indicating superior generalizability. Calibration analysis confirmed AutoML's predictive accuracy with the lowest Brier score (0.125) (Figure 3D).

### 3.4 Test set prediction validation

The AutoML model demonstrated robust predictive performance in independent validation, achieving an ROC-AUC of 0.8911 and PR-AUC of 0.8790 as illustrated in Figures 4A,B. Decision Curve Analysis across the clinical relevant threshold probability spectrum (1%-99%) revealed its superior net benefit compared to alternative approaches (Figure 4C). Calibration metrics confirmed excellent agreement between predicted probabilities and observed outcomes, with AutoML achieving the optimal Brier score (0.136) among all evaluated models (Figure 4D), indicative of its superior probability estimation accuracy. Comprehensive performance comparisons are presented in Table 4. Regarding calibration statistics, AutoML showed excellent calibration with Hosmer-Lemeshow *χ*^2^ = 3.29 (*p* = 0.915) and Spiegelhalter z = 0.36 (*p* = 0.721), outperforming other models—Logistic Regression (HL *χ*^2^ = 15.17, *p* = 0.056; z = 0.24, *p* = 0.814), Support Vector Machine (HL *χ*^2^ = 25.92, *p* = 0.001; z = 0.51, *p* = 0.609), AdaBoost (HL *χ*^2^ = 8.13, *p* = 0.421; z = 0.30, *p* = 0.766), XGBoost (HL *χ*^2^ = 11.41, *p* = 0.180; z = 0.30, *p* = 0.763), and LightGBM (HL *χ*^2^ = 4.90, *p* = 0.769; z = 0.30, *p* = 0.764). These quantitative results collectively demonstrate AutoML's superior calibration performance while maintaining high discriminative ability.

**Figure 4:**
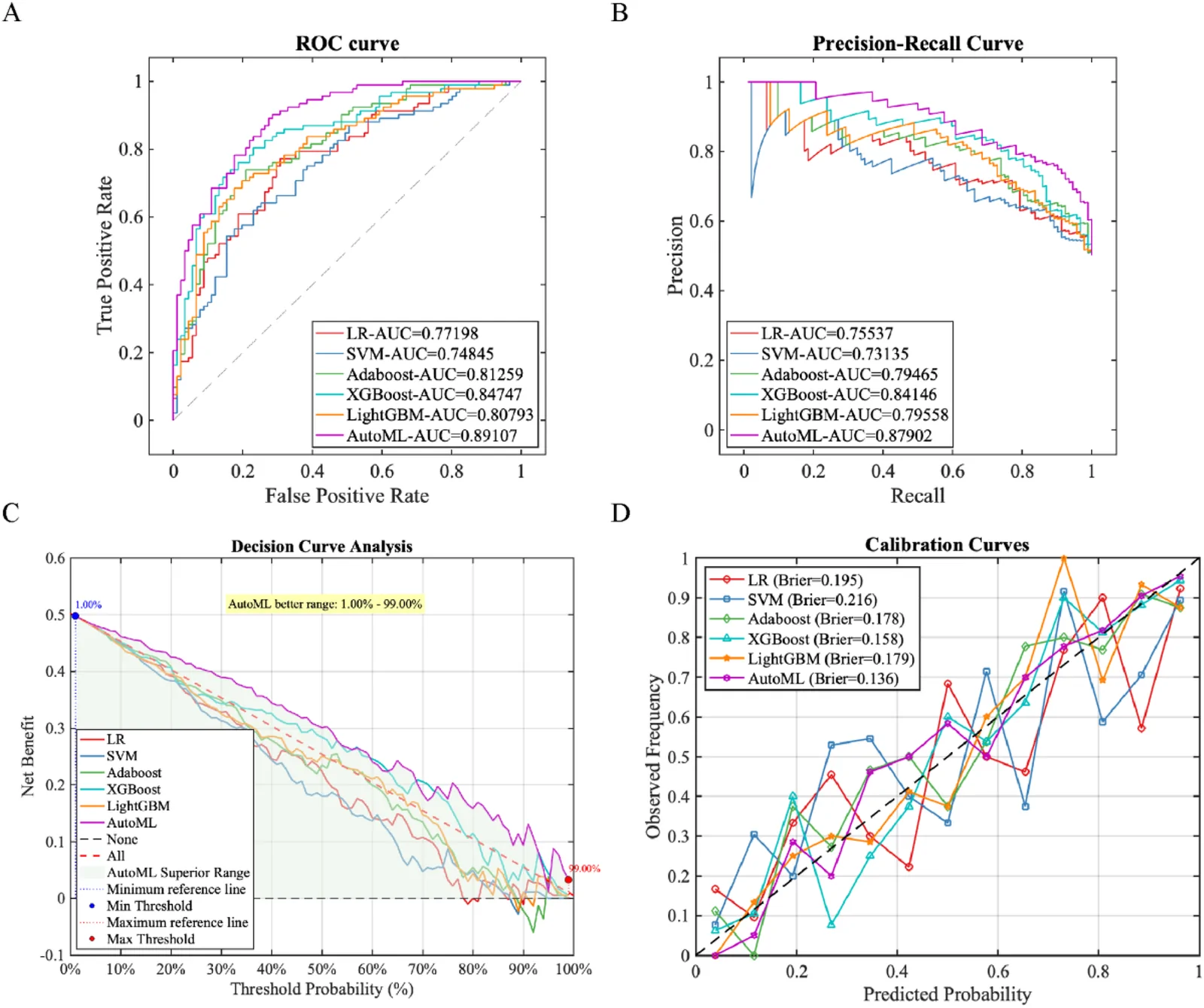
Test set prediction performance. **(A)** ROC curves; **(B)** PR curves; **(C)** DCA curves; **(D)** Calibration curves.

**Table 4:** Prediction efficacy metrics on the testing set.

Models	PRE	SEN	SPE	ACC	F1	ROC-AUC	PR-AUC
LR	0.7172	0.7717	0.6923	0.7322	0.7435	0.7720	0.7554
SVM	0.6349	0.8696	0.4945	0.6831	0.7339	0.7485	0.7314
Adaboost	0.7816	0.7391	0.7912	0.7650	0.7598	0.8126	0.7947
XGBoost	0.7789	0.8043	0.7692	0.7869	0.7914	0.8475	0.8415
LightGBM	0.6875	0.8370	0.6154	0.7268	0.7549	0.8079	0.7956
AutoML	0.7615	0.9022	0.7143	0.8087	0.8259	0.8911	0.8790

### 3.5 Optimizer ablation experiment

To evaluate the impact of different inner-layer optimization strategies, under the condition that the remaining settings of the AutoML pipeline (nested cross-validation, SMOTE, candidate feature space, data partitioning, and base learners) remained unchanged, IRFO was replaced with grid search, random search, and Bayesian optimization respectively to construct control models. On the independent test set (*n* = 183), the prediction efficacy of different inner-layer optimization strategies is shown in Table 5. The ROC-AUC and PR-AUC of IRFO-AutoML were 0.8911 and 0.8790, respectively; the ROC-AUC values for Bayesian optimization, random search, and grid search were 0.8718, 0.8643, and 0.8567, respectively, and the PR-AUC values were 0.8586, 0.8510, and 0.8435, respectively.

**Table 5:** Prediction efficacy metrics of the optimizer ablation experiment.

Model	PRE	SEN	SPE	ACC	F1	ROC-AUC	PR-AUC
IRFO-AutoML	0.7615	0.9022	0.7143	0.8087	0.8259	0.8911	0.8790
Bayesian optimization	0.7461	0.8713	0.7375	0.7913	0.8038	0.8718	0.8586
Random search	0.7401	0.8593	0.7465	0.7846	0.7952	0.8643	0.8510
Grid search	0.7340	0.8472	0.7556	0.7777	0.7865	0.8567	0.8435

### 3.6 Comparative analysis of prognostic prediction performance against traditional scoring systems

For the test set, the established AutoML model demonstrated significantly superior predictive performance compared to conventional scores (SOFA, APACHE II, and qSOFA), highlighting substantially enhanced predictive accuracy. These results are visually depicted in Figure 5.

**Figure 5:**
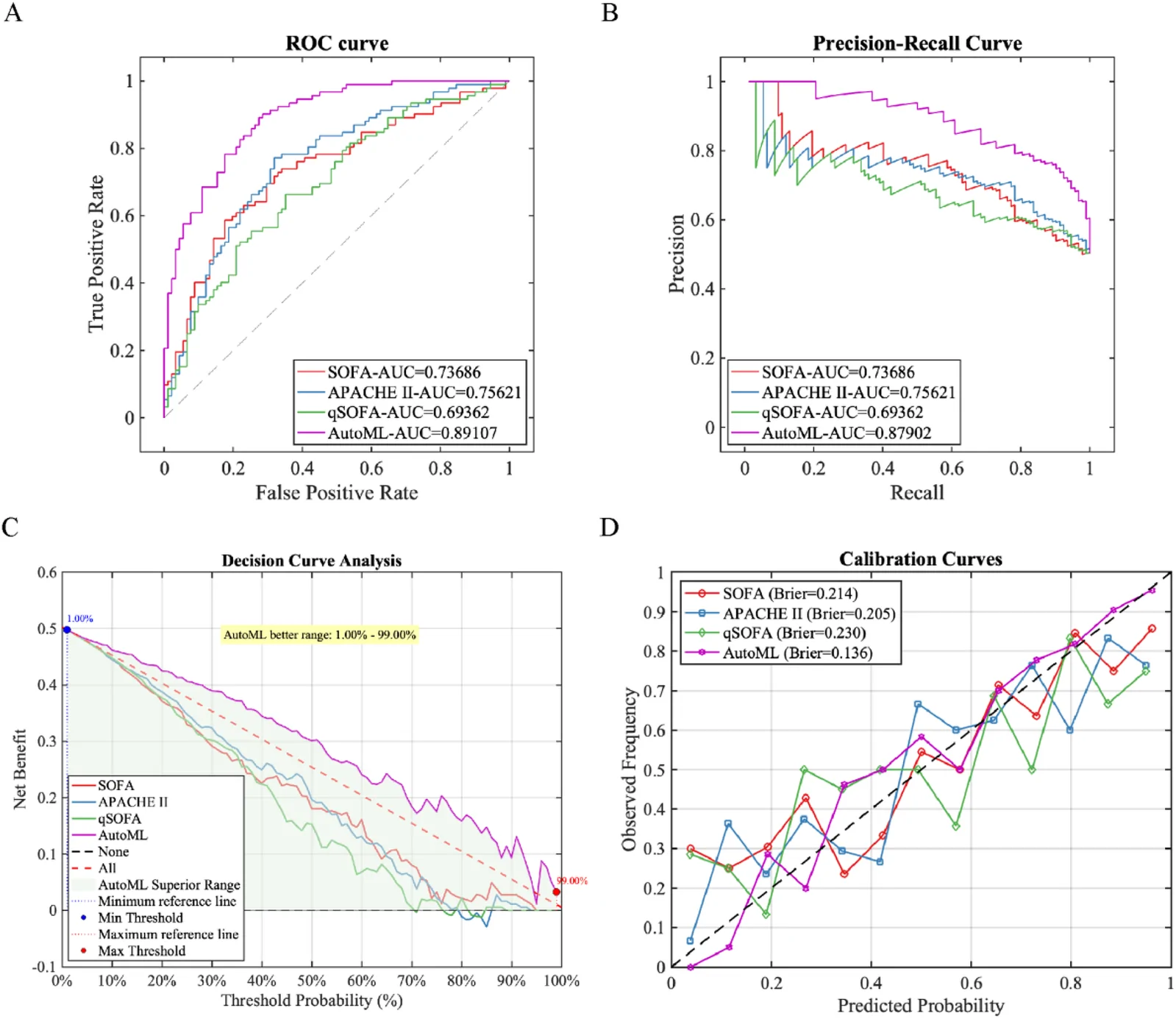
Prognostic performance comparison of traditional scoring systems. Note: **(A)** ROC curves; **(B)** PR curves; **(C)** DCA curves; **(D)** Calibration curves.

### 3.7 Temporal validation

The original cohort included 913 patients, and the additional temporal validation cohort included 169 patients consecutively admitted from February to December 2025, yielding a total of 1082 patients across the two phases. There were no statistically significant differences between the original cohort and the additional temporal validation cohort in terms of Age, NLR, MPV, Hb, PCT, D-dimer, and CRP (all *P* > 0.05), indicating that the two cohorts were generally comparable with respect to the baseline indicators relevant to the selected model. See Table 6 for details.

**Table 6:** Comparison of key features between the temporal validation cohort and the original cohort [median (Q_1_, Q_3_)].

Variable	Original Cohort (*n* = 913)	Temporal Validation Cohort (*n* = 169)	Z	*P*-value
Age (years)	67 (59, 75)	68 (59, 76)	−0.708	0.479
NLR	12.7 (8.3, 17.9)	13.0 (8.4, 18.5)	−0.493	0.622
MPV (fL)	10.7 (9.8, 11.6)	10.8 (9.9, 11.7)	−1.049	0.294
Hb (g/L)	100 (88, 114)	99 (87, 113)	0.799	0.424
PCT	7.7 (2.9, 16.4)	8.1 (3.0, 16.9)	−0.384	0.701
D-dimer	2130 (1169, 4061)	2200 (1205, 4181)	−0.383	0.702
CRP (mg/L)	94.4 (52.8, 151.1)	98.0 (54.2, 155.5)	−0.438	0.662

In the additional same-hospital temporal validation cohort, a total of 169 patients were included, among whom 85 (50.3%) died within 28 days. The frozen AutoML model achieved a ROC-AUC of 0.861 (95% CI 0.799–0.912) and a PR-AUC of 0.848 (95% CI 0.763–0.919). At the preset threshold of 0.50, the sensitivity was 0.847, specificity was 0.750, accuracy was 0.799, positive predictive value was 0.774, negative predictive value was 0.829, F1 score was 0.809, and Brier score was 0.153. See Figure 6.

**Figure 6:**
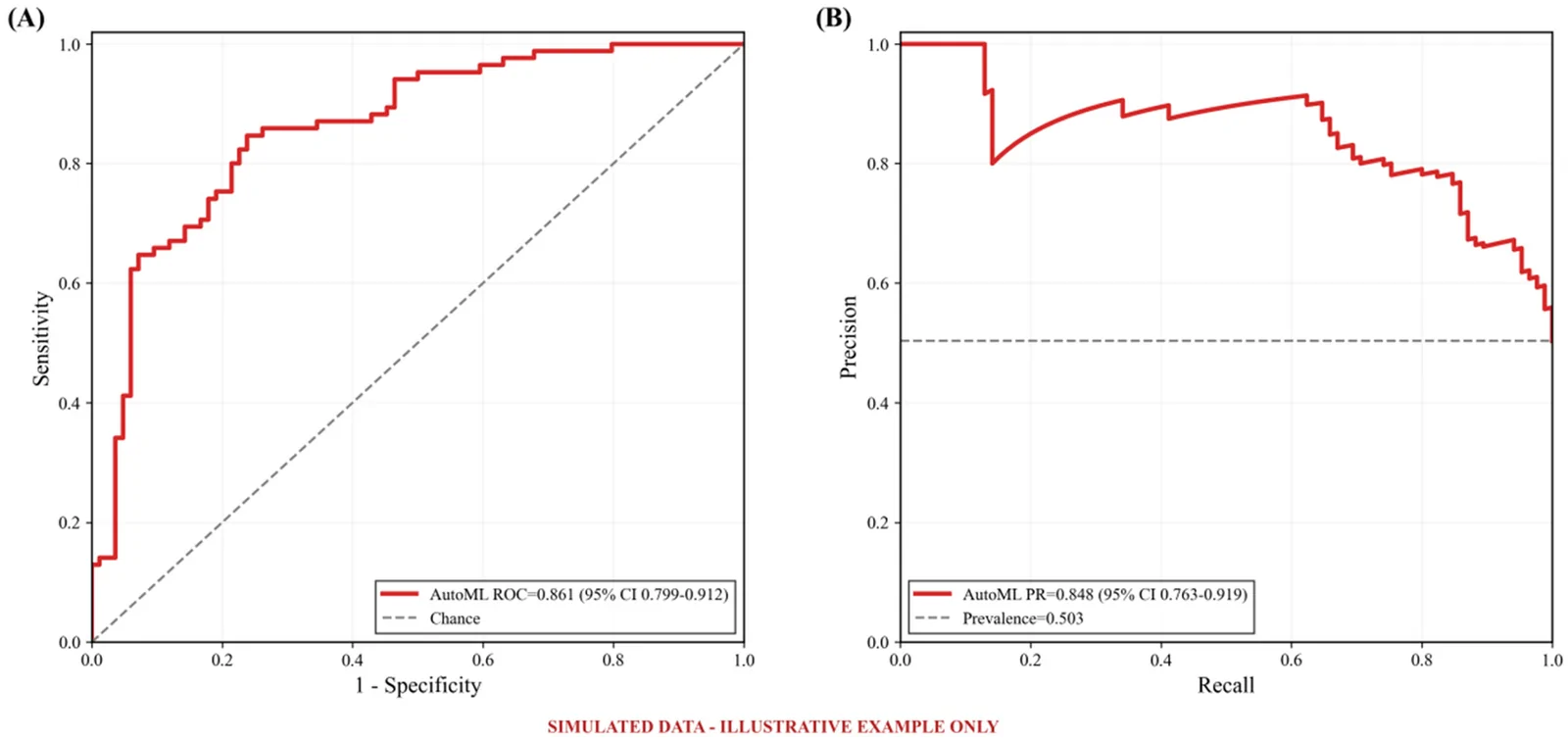
Temporal validation prediction results. **(A)** ROC curve; **(B)** PR curve.

### 3.8 Interpretability analysis

#### 3.8.1 Lasso regression

LASSO regression was implemented to perform feature selection on the training set data (Figure 7), validating the effectiveness of the AutoML model's feature screening. LASSO identified variables falling within one standard error of the minimum mean squared error (MSE) under the sparse model criterion (Lambda1SE), ultimately selecting seven key predictors: Age, NLR, MPV, Hb, PCT, D-dimer, and CRP. Importantly, this selection encompassed all features originally identified by the AutoML framework.

**Figure 7:**
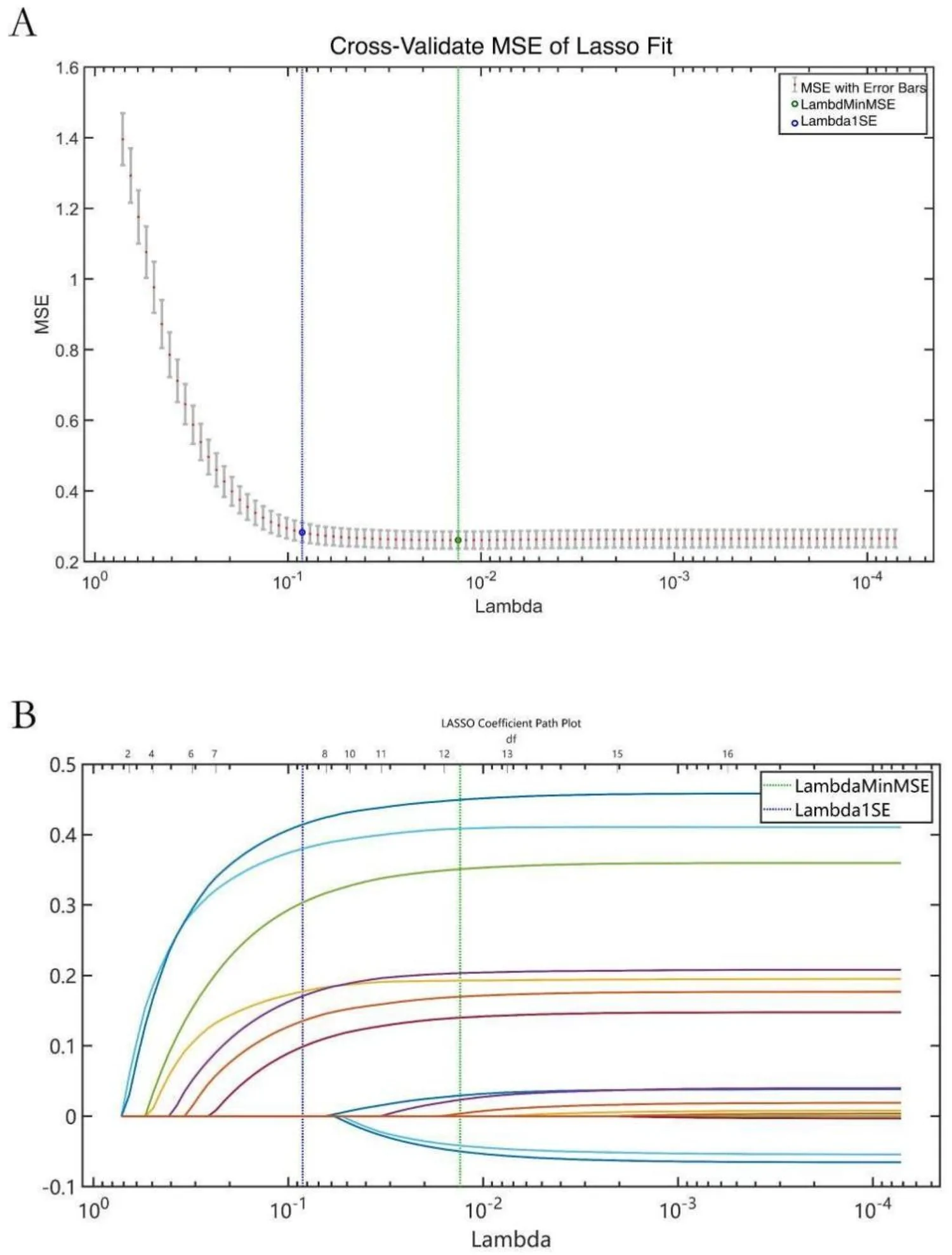
Lasso regression results. **(A)** Coefficient trajectories; **(B)** Cross-validation diagnostic plot.

#### 3.8.2 Shap interpretability analysis

According to the SHAP analysis results (Figures 8A,B), the importance ranking is as follows: 1. NLR; 2. Hb; 3. PCT; 4. D-dimer; 5. MPV; 6. Age. By comparing the decision paths of patients at different risk levels (Figure 8F), systematic differences in feature combinations between high-risk and low-risk patients can be identified. The decision path of high-risk patients is markedly shifted to the right, indicating the combined effect of multiple high-risk features.

**Figure 8:**
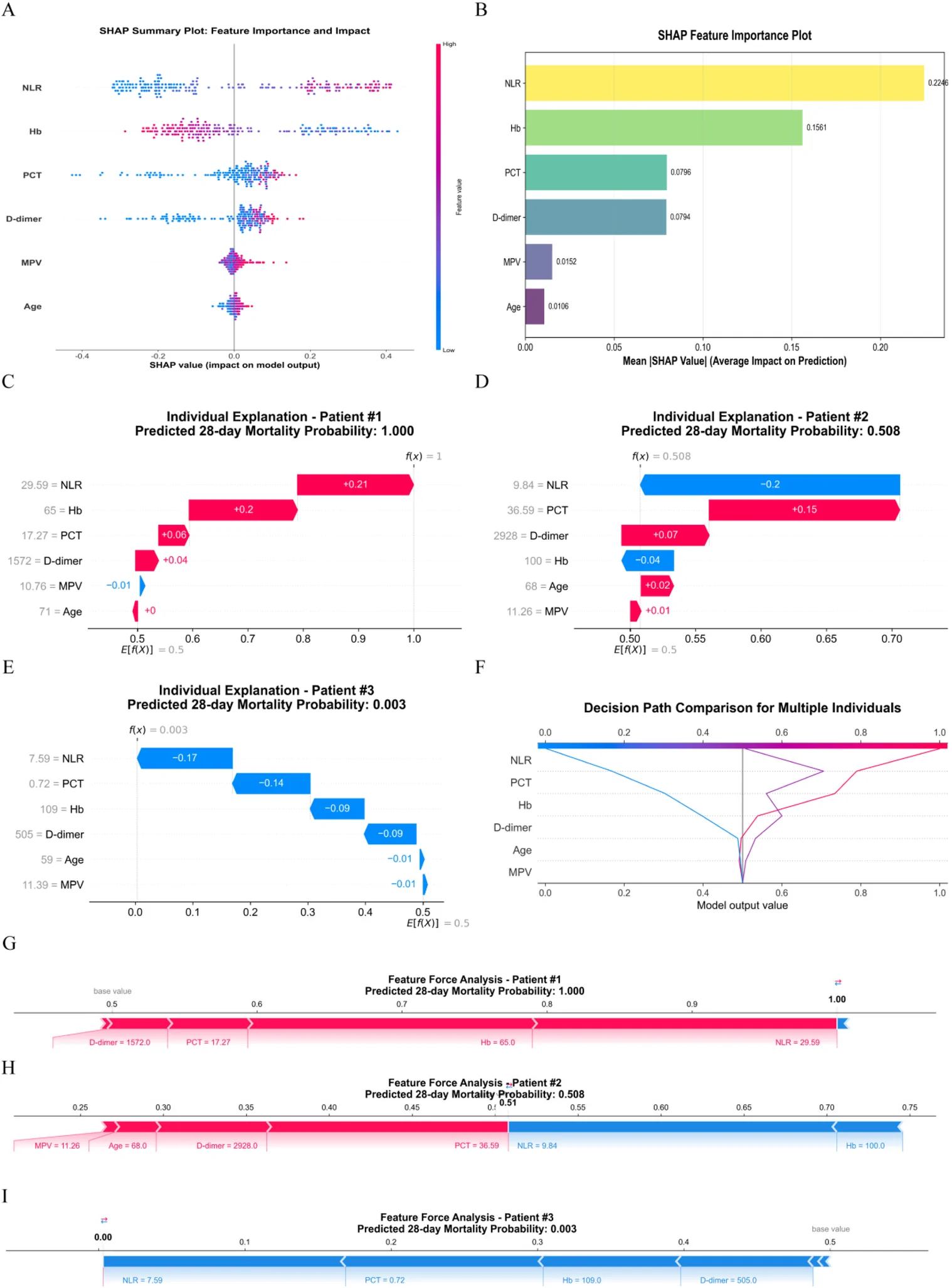
Shap interpretability analysis. **(A)** The Shapley summary plot comprehensively presents the overall impact patterns of various features on model predictions across all samples. Each point in the plot represents a feature and its SHAP value (i.e., the feature's contribution to prediction) for a specific sample. The color of the points indicates the actual value magnitude of the feature, while the distribution along the horizontal axis (SHAP values) reflects how feature values influence predictions (positive values increase predictions, negative values decrease them). This visualization allows intuitive identification of which features generally correlate with increases or decreases in predicted values, as well as trend relationships between feature influence and feature magnitudes; **(B)** The Shapley feature importance plot displays the overall importance ranking of each feature's impact on model predictions in bar chart form. Feature importance is determined by calculating the mean absolute SHAP value for each feature across all samples, thereby measuring its average contribution to model output variations. Longer bars indicate greater influence of the feature in the model's overall decision-making process, providing researchers with clear insight into the most critical factors driving predictions; **(C–E)** Waterfall plots illustrate the cumulative contribution process of each feature to individual patient predictions. The baseline value represents the model's average prediction for all patients, while feature contributions show how each feature affects the final prediction (red indicating increased risk, blue indicating decreased risk). The sum of all feature contributions yields the final predicted value; **(F)** The decision path plot compares decision pathways across multiple patients, demonstrating how different feature combinations lead to varying prediction outcomes. The horizontal axis shows predicted probabilities, the vertical axis lists features, and the curved pathways trace decision routes from baseline values to final predictions; **(G–I)** Force plots visually demonstrate how each feature “pushes” predictions toward higher or lower risk directions. Red arrows indicate features pushing predictions toward higher risk, blue arrows indicate features pushing toward lower risk, with arrow length representing the magnitude of influence.

High-risk patient (Figures 8C,G): NLR 29.59, Hb 65 g/L, PCT 17.27 g/L, D-dimer 1572 μg/L, MPV 10.76 fL, Age 71 years. Predicted 28-day mortality: 99.6%. Main risk factors: NLR (SHAP≈+0.21) > Hb (SHAP≈+0.20) > PCT (SHAP≈+0.06) > D-dimer (SHAP≈+0.04). This patient simultaneously met the high-risk interaction condition of NLR > 15 and Hb < 90 g/L, with MPV and Age contributing minimally (|SHAP|<0.01). Core conclusion: Markedly elevated NLR combined with decreased Hb is the primary source of high risk, suggesting that synergistic inflammation-anemia deterioration drives the extremely high mortality risk.

Moderate-risk patient (Figures 8D,H): NLR 9.84, Hb 100 g/L, PCT 36.59 g/L, D-dimer 2928 μg/L, MPV 11.26 fL, Age 68 years. Predicted 28-day mortality: 50.8%. The contributions of each factor are relatively balanced with opposing directions: PCT (SHAP≈+0.15) and D-dimer (SHAP≈+0.07) increase the risk, NLR (SHAP≈-0.20) and Hb (SHAP≈-0.04) decrease the risk, while Age (SHAP≈+0.03) and MPV (SHAP≈+0.01) have smaller effects. Core conclusion: The risk is at a critical level, with risk-elevating and risk-lowering factors counteracting each other; no single feature is absolutely dominant, and a comprehensive assessment is required.

Low-risk patient (Figures 8E,I): NLR 7.59, Hb 109 g/L, PCT 0.72 g/L, D-dimer 505 μg/L, MPV 11.39 fL, Age 59 years. Predicted 28-day mortality: 0.3%. Main protective factors: NLR (SHAP≈-0.17) > PCT (SHAP≈-0.14) > Hb (SHAP≈-0.09) > D-dimer (SHAP≈-0.09), while Age and MPV have minimal effects (|SHAP|<0.01). Core conclusion: The low risk originates from a protective combination in which NLR, PCT, Hb, and D-dimer are all at relatively favorable levels, with inflammation, infection, and coagulation indicators being generally low.

### 3.9 Decision threshold analysis and clinical implementation

The AutoML model constructed in this study outputs a continuous 28-day mortality risk probability for each patient. Converting continuous probabilities into binary classifications (high-risk/low-risk) requires setting a decision threshold. All model performance metrics reported in Table 4—including SEN, SPE, and ACC—were calculated using the conventional default threshold of 0.5. At this standard threshold, the AutoML model achieved optimal predictive performance on the test set (ACC: 0.8087, SEN: 0.9022, SPE: 0.7143).

However, in emergency septic shock management, clinical decision-making prioritizes minimizing missed diagnoses (i.e., maximizing sensitivity) over balanced statistical accuracy, as failure to identify high-risk patients carries severe consequences. We systematically analyzed the impact of adjusted decision thresholds on AutoML classification performance (Table 7). As shown in Table 7, lowering the threshold from 0.5 to 0.35 significantly increased sensitivity to 0.951, enabling detection of >95% of eventual fatalities and meeting the clinical need for “higher false alarms over missed cases”. This gain came at the cost of reduced specificity (0.648), resulting in misclassification of actual survivors as high-risk. Conversely, raising the threshold to 0.65 improved specificity to 0.890 but lowered sensitivity to 0.659, failing to detect >30% of fatal cases.

**Table 7:** Classification performance of the autoML model at different decision thresholds in the testing set.

Threshold of risk	SEN	SPE	PPV	NPV	ACC	F1
0.25	0.978	0.571	0.695	0.976	0.776	0.813
0.35 (Clinical Recommendation)	0.951	0.648	0.731	0.938	0.798	0.826
0.45	0.915	0.692	0.748	0.889	0.803	0.823
0.50 (Table 3 Baseline)	0.902	0.714	0.762	0.879	0.809	0.826
0.55	0.841	0.769	0.780	0.835	0.805	0.810
0.65	0.659	0.890	0.861	0.713	0.775	0.745

Based on this analysis, we propose a two-stage clinical decision support framework tailored to emergency settings: High-Risk Alert Tier (predicted risk ≥0.35): Triggers maximum-level alerts upon surpassing this threshold. Clinicians should complete all Sepsis Bundle interventions within one hour—including blood cultures, broad-spectrum antibiotics, hemodynamic monitoring, and goal-directed fluid resuscitation—with ICU admission priority. Enhanced Monitoring Tier (predicted risk <0.35): Patients admitted to emergency observation/ward units with continuous assessments at 6-, 12-, and 24-hour intervals using sequential model-driven risk re-evaluations.

This framework transforms singular probability outputs into actionable clinical pathways. By setting the threshold at 0.35, it prioritizes early detection breadth (high sensitivity) while managing false positives through dynamic monitoring, thereby optimizing risk stratification efficiency under limited medical resources.

### 3.10 Clinical decision support system

An interactive prediction interface was developed featuring real-time mortality probability calculation (0-100%). Clinicians input admission indicators via the feature panel (Figure 9), triggering instantaneous AutoML-based prognosis assessment to guide therapeutic stratification. To facilitate reproducibility and dissemination, the complete technical description of the clinical decision support system interface—including its overall architecture, functional modules (feature entry panel, real-time risk calculation engine, risk stratification and alert output), input and output specifications, and operating environment—has been described in detail in Supplementary Material 2.

**Figure 9:**
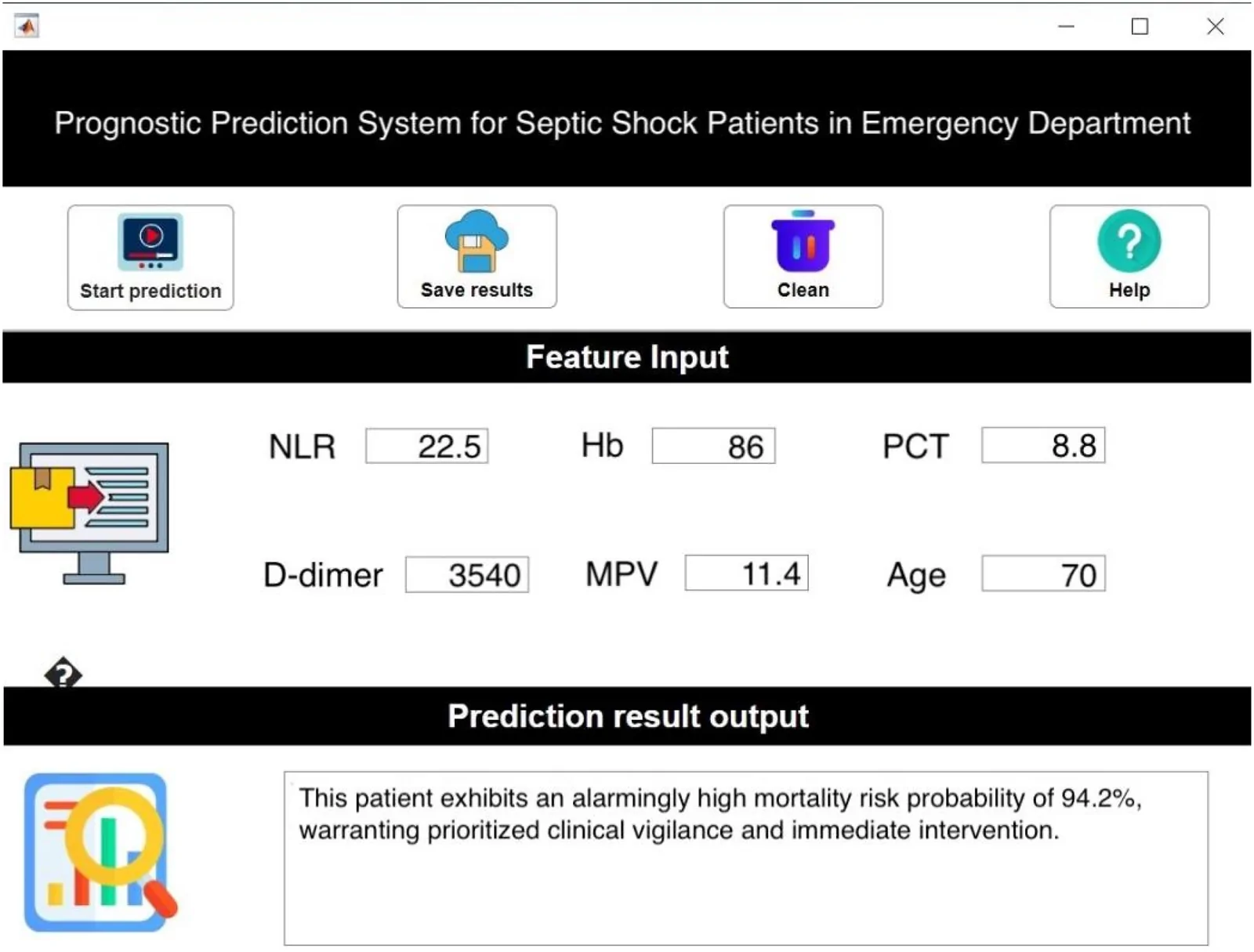
Demonstration of the clinical decision support system.

## 4 Discussion

This study established an AutoML-based predictive framework for 28-day mortality risk in emergency septic shock patients, leveraging retrospective analysis of 913 clinical cases to develop an integrated approach empowered by the Improved Rüppell's Fox Optimizer (IRFO). This novel methodology achieved simultaneous feature selection and hyperparameter optimization, demonstrating superior predictive performance over conventional models like logistic regression and support vector machines during independent testing. Comprehensive metrics confirmed the model's robust discriminative capacity, while SHAP interpretability analysis revealed critical interaction pathways among predictors. The deployment of a real-time interactive decision interface further enhances clinical utility by providing dynamic mortality probability calculations to support emergency interventions. Key innovations include the IRFO algorithm's application to medical prediction through chaotic mapping-enhanced initial population distribution and adaptive Lévy flight steps, effectively addressing metaheuristic algorithms’ local optimum limitations in feature selection. The dual-stage AutoML optimization framework successfully uncovered complex nonlinear relationships within routine blood biomarkers, capturing patterns that traditional linear models may not fully represent. Crucially, the tight integration of explainability and clinical application manifests through SHAP's identification of globally significant features (notably NLR) and high-stakes interaction patterns, augmented by decision curve analysis validating substantial net clinical benefit at critical risk thresholds—suggesting potential clinical utility that requires confirmation in prospective, multicenter studies.

Traditional prognostic tools like SOFA, APACHE II, and qSOFA rely on static single-timepoint assessments that fail to capture sepsis’ dynamic immune-coagulation network. While SOFA remains widely adopted, its fixed-weight architecture cannot model nonlinear interactions between biomarkers like NLR and D-dimer, resulting in suboptimal early prognostic accuracy in emergency settings (18). Contemporary biomarker-driven approaches using PCT or Hb show promise yet suffer from insufficient feature extraction, overlooking readily available indicators such as systemic immune-inflammation indices. Although some studies have attempted SOFA augmentation with immunoinflammatory metrics, these models often rely on fixed formulas and lack an efficient, real-time computational framework for individualized bedside risk assessment. A key contribution of this study is AutoML's capacity to process high-dimensional heterogeneous data, enabling automated identification of relevant feature combinations from routine admission biomarkers. Compared to prior machine learning implementations (e.g., multivariate models based on systemic inflammation indices), our AutoML-driven feature subset automation reduces manual intervention, with favorable AUC performance on the independent test set suggesting potential utility in time-sensitive emergency settings.

Notably, the six variables visualized in the SHAP analysis represent the complete final feature set selected by the IRFO-AutoML pipeline, rather than a manually chosen subset. These six indicators therefore constitute all model inputs used for prediction, and no additional candidate variables were omitted from the visualization. It should be acknowledged, however, that SHAP values reflect model-conditional contributions within the current feature set and dataset, and their importance ranking may vary with the number of included indicators, sample size, and correlations among features. Therefore, the SHAP-based importance should not be interpreted as independent clinical effect sizes or causal importance. Pathophysiological contextualization remains essential when interpreting key predictors elucidated by SHAP's game-theoretic quantification. The primacy of NLR reflects neutrophil-lymphocyte ratio dysregulation signifying systemic hyperinflammation coupled with evolving immunoparalysis, whereby neutrophilia drives organ damage via NETosis while lymphopenia denotes adaptive immune exhaustion—directly linking immune dysfunction to multi-organ failure; values exceeding physiological ranges underscore acute inflammatory burden as pivotal in mortality prediction, consistent with established sepsis-prognosis correlations (19, 20). Recent evidence confirms that modulation of this dysregulated innate/adaptive immunity continuum may improve outcomes (21). Hemoglobin (Hb) emerged as the secondary determinant, its reduction resulting not only from hemorrhage/anemia but also inflammation-driven erythropoiesis suppression and impaired erythrocyte function, exacerbating tissue hypoxia through impaired microcirculatory flow and oxygen delivery—forming a positive feedback loop with shock progression (22–24). Critically, SHAP interaction mapping revealed Hb deficiency potentiates NLR's inflammatory impact via amplified hypoxic stress (24). The tertiary predictor PCT serves as a broad-spectrum infection marker whose elevation indicates bacterial toxin load, yet despite high diagnostic specificity, it proves inadequate as a standalone prognostic indicator (25). Complementary PCT integration with inflammatory markers (e.g., NLR, IL-6) substantially enhances early risk stratification (26), while concomitant D-dimer elevation signals dysregulated immunothrombosis via sepsis’ coagulopathy-endothelial dysfunction crosstalk, indicative of microvascular thrombosis (27). Joint PCT/D-dimer assessment significantly improves severity stratification by capturing concurrent hyperinflammation and coagulopathy—corroborated by our synergistic mortality escalation finding (28). Though secondary in impact, MPV's pathological increase indicates accelerated platelet turnover during thrombocytopenia, linking platelet hyperactivation with endothelial damage, wherein persistent thrombocytopenia powerfully predicts outcomes (29, 30). Collectively, these mechanistic interpretations support the model's consistency with septic shock's known “triad dysregulation” of immune-metabolic-coagulation pathways. Recent studies have proposed composite indices such as the ILR and the LIP score (31, 32), as well as the SMART risk stratification system (33), which may provide complementary prognostic information beyond the routine laboratory features used in the present model. These emerging tools were not incorporated into the current single-center retrospective analysis, and whether they offer incremental value over the six-feature AutoML model should be evaluated in prospective multicenter cohorts with identical endpoints. The SMART system, in particular, may target a different clinical purpose than 28-day mortality prediction in septic shock, and therefore is better regarded as a complementary tool rather than a direct replacement for the present model.

From a clinical implementation perspective, the proposed two-stage framework is designed to be embedded within the existing emergency department workflow rather than functioning as a standalone tool. First, the prediction model can be deployed as a containerized web service or a lightweight FHIR/CDS Hooks application that automatically retrieves the same admission laboratory indicators used for model development and returns the predicted risk score together with the corresponding feature contributions to the electronic health record clinician workstation. This integration avoids manual data entry and minimizes disruption to clinical documentation. Second, the high-risk alert tier is intended to align with the Sepsis Bundle pathway: when the predicted risk exceeds the predefined threshold, the system can trigger a high-priority alert prompting the treating team to complete blood cultures, initiate broad-spectrum antibiotics, and perform hemodynamic assessment within the first hour. The enhanced monitoring tier can be integrated with routine reassessment time points in the emergency observation unit or ward, supporting sequential risk re-evaluation at scheduled intervals. Several barriers to adoption should be acknowledged. Alert fatigue is a major concern in busy emergency departments; threshold-based two-tier alerting and adjustable risk cut-offs may help reduce unnecessary interruptions by reserving the highest-priority alerts for patients with the greatest predicted risk. IT infrastructure limitations, including interoperability with hospital information systems, real-time data availability, downtime management, and model versioning, may also influence implementation. In addition, clinician acceptance depends not only on predictive performance but also on interpretability; therefore, explainable outputs that highlight the most influential variables may improve trust and facilitate integration into clinical decision-making.

Several limitations warrant acknowledgment: The single-center retrospective design, though substantial (*n* = 913), risks regional selection bias, while stringent exclusions (e.g., pre-transfusion cases to preserve Hb integrity) omitted clinical subgroups like trauma patients. Furthermore, the reliance on single-center retrospective data with internal validation via random dataset splitting may overestimate model performance due to potential temporal confounding and unmeasured center-specific biases. While stratified randomization ensured comparable training/testing distributions, this internal balance should not obscure a more fundamental sampling issue: the retrospective cohort itself comprised approximately equal proportions of 28-day survivors and non-survivors. Specifically, the overall 28-day mortality was 50.2% (458/913), with 50.3% and 49.7% in the training and testing sets, respectively. This near 1:1 death/survival composition reflects the retrospective sample selection strategy, which tended to balance deceased and surviving patients, and it differs substantially from the approximately 23% 28-day mortality observed in real-world clinical practice. Although such balanced selection improves statistical power for identifying mortality-related features, it may introduce spectrum bias, alter the pretest probability of death, and reduce the generalizability of the reported AUC, calibration, and threshold-based metrics to an unselected clinical septic shock population. Temporal validation (e.g., training on earlier cases, testing on later admissions) or multi-internal/external validation would more rigorously assess chronological robustness and generalizability across evolving clinical practices. Accordingly, the reported test-set AUC of 0.891 should be interpreted as an upper-bound estimate, as performance can be inflated by this single-random-split design. Importantly, because this remains a single-center retrospective analysis, the true performance in different healthcare settings—including different countries, community versus tertiary hospitals, and different patient demographics—remains unknown, and the current AUC should therefore not be generalized beyond this internal validation context. A prospective multi-center external validation study is currently underway and will be reported in a subsequent publication to confirm the model's robustness. Future iterations will undertake prospective multi-center validation to mitigate these limitations. Static admission data cannot capture sepsis’ temporal dynamics, limiting real-time intervention assessment. In addition, because the model relies solely on single-time-point admission data, it is not intended to guide continuous therapeutic decisions that require repeated, time-varying measurements—such as antibiotic de-escalation or fluid responsiveness assessment. The feature set overlooked parameters from conventional scores (e.g., organ oxygenation markers), while AutoML's computational intensity challenges resource-constrained emergency deployments. A key limitation is that the current model is a static, admission-based predictor that utilizes biomarkers from a single time point rather than tracking longitudinal disease evolution. This design prioritizes rapid risk stratification during critical early resuscitation phases. Regarding the suggestion of time-to-event analysis, we agree that constructing survival models (e.g., Cox proportional hazards models) for serial biomarker trajectories would more faithfully characterize the temporal evolution of sepsis; however, the data in this study comprise a binary 28-day mortality outcome, with biomarkers collected only at a single admission time point and lacking precise event times and serial measurements, so this cannot be achieved in the current dataset. Such time-to-event and serial trajectory modeling has been explicitly designated as a priority direction for the ongoing prospective multicenter cohort. Future investigations should: (1) Establish multicentered prospective validation; (2) Incorporate dynamic monitoring, subsequent investigations will incorporate dynamic monitoring of inflammatory and thrombotic markers through serial measurements of procalcitonin, lactate, neutrophil-lymphocyte ratio, and mean platelet volume at 0/24/72-hour intervals. This trajectory aligns with the emerging frontier of continuous biomarker monitoring, where advances in microneedle-based interstitial-fluid biosensors and wearable vital-sign integration are beginning to make real-time, trajectory-based prognostic modeling feasible at the point of care. Situated within this evolving landscape, the present static admission-based model provides a foundational framework upon which future continuous-monitoring systems can be built, ultimately enabling the transition from single-timepoint risk stratification to dynamic, trajectory-informed clinical decision-making. (3) Fuse AutoML with novel algorithms (e.g., deep learning) for temporal feature processing; (4) Develop mobile decision tools for primary care; (5) Extend the framework to sepsis-related syndromes (e.g., multiorgan dysfunction); and (6) Conduct randomized trials evaluating survival impact and cost-efficacy when integrated into clinical workflows; (7) Because the current CDSS has not yet been prospectively deployed in a real EHR environment, future work should evaluate its performance and usability through prospective implementation studies and, ultimately, cluster-randomized or stepped-wedge trials comparing standard care with CDSS-assisted care in the emergency department.

In conclusion, IRFO-optimized AutoML effectively harnesses routine hematological biomarkers to establish an explainable prognostic system for emergency septic shock. Its capacity for complex, nonlinear modeling outperforms traditional methods in accuracy and, combined with the prospective integration of dynamic, longitudinal data poised to transform therapeutic outcomes. This framework's pivotal innovation—reconciling computational efficiency with clinical interpretability—charts new pathways for overcoming sepsis mortality prediction barriers, with prospective dynamic data integration poised to transform therapeutic outcomes.

## Data Availability

The raw data supporting the conclusions of this article will be made available by the authors, without undue reservation.
