# Supplementary material for: Development of an automated machine learning-based prognostic prediction model for emergency septic shock patients
Source: Front Med (Lausanne). 2026 Oct 26;13:1860425. doi: 10.3389/fmed.2026.1860425 (PMC13745730; doi:10.3389/fmed.2026.1860425)
Supplement: Supplementary file 1 [file Table1.docx]

**Algorithm 1: IRFO-optimized AutoML Feature Selection and Hyperparameter Tuning**

%% 1. Initialization

Set parameters: pop_size, max_iter, lb, ub, dim

% Chaotic mapping initialization for population diversity

for i = 1:pop_size

for d = 1:dim

chaotic_val = logistic_map(rand()); % Generate chaotic sequence

population(i, d) = lb(d) + chaotic_val * (ub(d) - lb(d));

end

end

%% 2. Main Optimization Loop

for iter = 1:max_iter

for i = 1:pop_size

% 2.1 Feature Subset & Hyperparameter Decoding

features = bin2dec(population(i, 1:num_features)); % Discrete: Feature Selection

hyperparams = population(i, num_features+1:end); % Continuous: Hyperparameters

% 2.2 Fitness Evaluation (Inner CV AUC)

fitness(i) = -crossval_model(features, hyperparams); % Minimize negative AUC

end

% 2.3 Sort and Identify Best Solution

[sorted_fit, idx] = sort(fitness);

alpha_pos = population(idx(1), :); % Best solution

% 2.4 Dynamic Lévy Flight Position Update

a = 2 - iter * (2 / max_iter); % Exploration rate, decays linearly

for i = 1:pop_size

r1 = rand(); r2 = rand();

A = 2 * a * r1 - a; % Coefficient vector

C = 2 * r2;

% Distance to Alpha

D = abs(C * alpha_pos - population(i, :));

X1 = alpha_pos - A * D; % Standard encircling mechanism

% Apply dynamic Lévy flight for balance

if rand() > 0.5

levy_step = levy_flight(dim);

new_pos = X1 + randn() .* levy_step; % Exploration

else

new_pos = X1; % Exploitation

end

% Bound checking and update if better

new_pos = boundary_check(new_pos, lb, ub);

if eval_fitness(new_pos) < fitness(i)

population(i, :) = new_pos;

end

end

end

%% 3. Return optimal feature subset and hyperparameters

**Supplementary Figure 1. Optimization performance comparison of population-based algorithms**

(Note: Boxplots display optimization results after 30 independent runs on CEC2022 functions, illustrating algorithm stability and robustness.)

**Supplementary Figure 2. Convergence performance comparison of population-based algorithms**

(Note: Convergence curves reflect optimization progress, highlighting convergence speed and local optimum avoidance capability.)
