# Supplementary material for: Development of an automated machine learning-based prognostic prediction model for emergency septic shock patients
Source: Front Med (Lausanne). 2026 Oct 26;13:1860425. doi: 10.3389/fmed.2026.1860425 (PMC13745730; doi:10.3389/fmed.2026.1860425)
Supplement: Supplementary file 2 [file Table2.docx]

**Supplementary Material 2**

**Clinical Decision Support System (CDSS): Detailed Interface Description**

**1. Overview**

The Clinical Decision Support System (CDSS) is an interactive prediction interface that accompanies the IRFO-optimized AutoML prognostic model. It provides clinicians with a real-time, bedside tool for estimating the 28-day all-cause mortality risk of emergency septic shock patients upon admission. By entering six routine admission biomarkers, the system immediately outputs a continuous mortality probability (0–100%) together with a risk-stratification recommendation, thereby supporting early risk assessment and triage decisions.

**2. System Architecture**

The CDSS is organized as a lightweight, modular pipeline comprising four logical layers:

(1) Input Layer — a graphical feature-entry panel for the six predictor variables;

(2) Inference Layer — the frozen IRFO-AutoML model (XGBoost/LightGBM base learners) that performs real-time prediction;

(3) Stratification Layer — a threshold-based rule that maps the predicted probability to a two-tier risk category; and

(4) Output Layer — display of the mortality probability and the corresponding clinical alert.

The system operates entirely offline on the six admission features and returns results instantly; no longitudinal or time-varying data are required at the point of prediction.

**3. Functional Modules**

**3.1 Feature Input Panel**

Clinicians enter the six model predictors measured at admission: NLR, Hb, PCT, D-dimer, MPV, and age. The panel enforces valid numeric ranges and units for each field (Table S2-1).

**3.2 Real-time Risk Calculation Engine**

Upon submission, the entered values are passed to the frozen IRFO-AutoML model. The engine applies the same preprocessing (feature subset and scaling) used during model development and returns the predicted probability of 28-day all-cause mortality. The computation is instantaneous (on the order of milliseconds), enabling bedside use.

**3.3 Risk Stratification and Alert Output**

The continuous probability is converted into a two-tier clinical recommendation using the operating threshold of 0.35:

• High-Risk Alert Tier (predicted risk ≥ 0.35): maximum-level alert; clinicians should complete all Sepsis Bundle interventions within one hour and prioritize ICU admission.

• Enhanced Monitoring Tier (predicted risk < 0.35): patients admitted to emergency observation/ward units with sequential re-evaluation at 6-, 12-, and 24-hour intervals.

**4. Input/Output Specification**

Table S2-1 lists the six input features with their units and data types.

***Table S2-1. CDSS input features***

| **Feature** | **Unit** | **Data type** |
| --- | --- | --- |
| NLR | — (ratio) | Positive numeric |
| Hb | g/L | Numeric |
| PCT | ng/mL | Numeric |
| D-dimer | μg/L | Numeric |
| MPV | fL | Numeric |
| Age | years | Integer |

**Output fields:**

• Mortality probability: a continuous value in [0, 1], displayed as a percentage (0–100%);

• Risk category: “High-Risk” or “Enhanced Monitoring”, derived from the 0.35 threshold.

**5. Runtime Environment and Deployment**

The inference engine was implemented within the same environment used for model development (MATLAB 2024a). The interface requires no network connectivity and can be deployed on standard desktop workstations in the emergency department. In future deployments, the system is intended to be integrated into the electronic health record (EHR) workflow.

**6. Typical Usage Workflow**

(1) Enter the six admission biomarker values; (2) trigger prediction; (3) read the mortality probability and risk category; and (4) follow the corresponding clinical pathway.
